# The impact of anxiety on postural control: CO_2_ challenge model

**DOI:** 10.1111/psyp.14192

**Published:** 2022-10-06

**Authors:** Alexander N. W. Taylor, Daniel C. Low, Gregory S. Walsh, Nigel Holt

**Affiliations:** ^1^ Department of Psychology Aberystwyth University Aberystwyth UK; ^2^ Centre for Human Performance, Exercise and Rehabilitation Brunel University London London UK; ^3^ Department of Sport, Health Sciences and Social Work Oxford Brookes University Oxford UK

**Keywords:** anxiety, balance control, CO_2_ challenge, heart rate, motor control, psychophysiology

## Abstract

Anxiety and balance and postural control are linked via common neural pathways, such as the parabrachial nucleus network. A laboratory‐based model of general anxiety disorder (GAD) using the CO_2_ challenge, has potential to be used to observe this relationship, potentially mimicking subjective, autonomic, and neuropsychological features of GAD. The current feasibility study used the CO_2_ challenge to explore postural control changes in healthy adults. It was predicted that during the CO_2_ condition, participants would show increased postural sway path length and decreased sway stability, compared with a normal air breathing condition. To assess this, heart and breathing rate, quiet standing postural sway path length, sway dynamic stability, and subjective measures of emotion were measured either before and after or during and after the inhalation conditions. Results demonstrated that CO_2_ inhalation led to both an increase in sway path length and reduced sway stability compared to the air breathing conditions; the effect on sway path lasted after the inhalation of CO_2_ had ceased. Additionally, replication of HR and subjective measures of emotion were observed when comparing air and CO_2_ conditions. This provides experimental evidence that CO_2_ inhalation can affect balance, suggestive of shared mechanisms between anxiety and balance performance, as well as indicating that the CO_2_ model of GAD is suitable to look at changes in balance performance in healthy adults. Future use of this model to explore factors that can reduce the influence of GAD on balance would be beneficial as would a more detailed exploration of the neural pathways associated with the associated comorbidity.

## Abstract 2

This feasibility study demonstrates the potential of using the CO_2_ challenge model as an experimental tool to better understand the relationship between anxiety and balance disorders. We evidenced increased path sway and stability as the result of 7.5% CO_2_ inhalation induced anxiety state. Furthermore, increased heart rate and poorer self‐reported mood state due to the CO_2_ inhalation, corroborate previous findings of generalised anxiety disorder symptomology in this model.

## 1 INTRODUCTION

Anxiety disorders are considered a major worldwide burden (Baxter et al., 2013; Fineberg et al., 2013; Santomauro et al., 2021), of which Generalized Anxiety Disorder (GAD) has been shown to be the most common (Remes et al., 2018). The cost of GAD and related anxiety disorders have been estimated to cost approximately $11.7 billion in direct and indirect costs in the UK (Fineberg et al., 2013). GAD is associated with reduced health‐related quality of life (HRQoL; Revicki et al., 2012), increased suicidality and unemployment, loss of productivity and self‐worth, and increased health‐care utilization (Khan et al., 2002; Olatunji et al., 2007). The symptoms of GAD tend to include persistent and excessive anxiety for a period of at least 6 months, along with constant worry and symptoms of restlessness and inability to concentrate and/or sleep (DeMartini et al., 2019).

Evidence indicates that a comorbidity exists between anxiety and balance performance and abilities (Bart et al., 2009; Bolmont et al., 2002; Kogan et al., 2008; Sklare et al., 2001; Sturnieks et al., 2016). Biomechanical study of this relationship has shown that those with high anxiety had impacted postural control, used to maintain a balanced standing state, whereby greater sway path length is observed (Stins et al., 2009). This is indicative of less postural stability (Johansson et al., 2017; Paillard & Noe, 2015; Walsh et al., 2021), which can also be demonstrated through use of non‐linear measures such as Lyapunov Exponent (LyE); which demonstrates speed of attraction (convergence) or divergence of trajectories in each dimension of an attractor, where in the case of balance a greater value suggests more instability (Smith et al., 2010). It has been demonstrated that the vestibulo‐recipient region of the parabrachial nucleus and the reciprocal connections with the extended central amygdaloid nucleus, infralimbic cortex, and hypothalamus, is important in the development of this comorbidity (Balaban, 2002; Balaban & Thayer, 2001). Consequently, when the activity of these limbic structures is excessive, anxiety and balance can be impacted simultaneously, (Stins et al., 2009). If this mechanism is correct, anxiety disorders should benefit to some extent from balance training (Stins et al., 2009), a suggestion supported by the research of Bart et al. (2009) with children.

The exploration of potentially novel treatments is aided by cost‐effective and timely use of experimental medicine models with healthy volunteers as participants (Baldwin et al., 2017). The CO_2_ challenge model is used to explore treatments for anxiety disorders such as GAD, mimicking the subjective, autonomic, and neuropsychological features (Bailey et al., 2007; Garner et al., 2011); it is also reactive to pharmacological and psychological treatment (Ainsworth et al., 2015; Bailey et al., 2007). Importantly, it has been demonstrated that increased functional connectivity between the ventromedial prefrontal cortex and the amygdala can be demonstrated through use of the CO_2_ challenge model (Huneke et al., 2020). This pathway is important when assessing threat (Mobbs et al., 2007), and importantly reduced functional connectivity between the prefrontal cortex and amygdala is observed during threat processing of GAD individuals (Monk et al., 2008). Given this, and the aforementioned links between brain regions underpinning the comorbidity, the model offers an approach for testing potential treatments for both GAD and balance performance at a ‘proof of concept’ stage (Redfern et al., 2007; Yardley & Redfern, 2001). Furthermore, using the CO_2_ model of anxiety (Bailey et al., 2005, 2007) in those with low trait anxiety, it can be used to explore the hypothesis of shared connections as the mechanism for the comorbidity.

The aim of the current study was to investigate the feasibility of using the CO_2_ challenge model as a means of exploring the anxiety and balance relationship. This was achieved in a sample of healthy younger adults, during normal air and CO_2_ challenge inhalation conditions, where changes in balance were observed in the context of other measures, such as heart and breathing rate, self‐reported trait and state measures of anxiety and emotion before and during the experimental conditions. Informed by previous literature (e.g., Bailey et al., 2007; Garner et al., 2011) which has observed only increases in anxiety symptomology due to the CO_2_ challenge, it was predicted that there will be increases in heart rate (HR), breathing rate (BR), and self‐report state symptoms of affect and anxiety in the CO_2_ compared to normal air post‐inhalation period and compared to pre‐inhalation air condition. Importantly, prior research also highlights mood states and anxiety results in poorer balance control (Bolmont et al., 2002), as well as other factors such as removal of visual information (Raffalt et al., 2019), increased age (Walsh, 2021) and disorders such as multiple sclerosis (Gera et al., 2020). Emphasizing a general decrease in balance control due to comorbid factors. Therefore, it is also predicted that there would be poorer balance control demonstrated by an increased postural sway and decreased sway stability in the CO_2_ versus normal air condition during the inhalation period but not during post‐inhalation condition.

## 2 METHOD

### 2.1 Participants

Ten healthy volunteers (*M:F* = 7:3) recruited from the general population were participants for this study (*M*_age_ = 21.7 years; *SD* = 2.5, range = 18–25). Each participant was offered financial reward in the form of an e‐voucher worth up to £20. Despite this incentive, participants were able to cease participation at any point, without consequence and were still given the reward.

Participants completed a pre‐study email consent and structured diagnostic screening telephone interview, to ensure their suitability for participation. This included questions on general physical and mental health (including the Mini International Neuropsychiatric Interview – MINI; Sheehan et al., 1998). From this, participants were excluded if they met the criteria of any anxiety‐related disorders e.g., GAD, social anxiety, PTSD. Additional exclusion criteria that is consistent with previous studies (e.g. Garner et al., 2012; Pinkney et al., 2014), included personal or family history of panic disorder or panic attacks, use of medication (past eight weeks bar apart from local treatment, occasional aspirin or paracetamol, and contraceptives), smoking, history of asthma/respiratory disease, diabetes, migraines, cardiovascular disease, excessive alcohol consumption (21 units/week for male and 14 unit/week for females) or a positive alcohol breath test on the day of testing, current or past alcohol or drug dependence (including recent recreational drug use), being under‐ or over‐weight (body mass index [BMI] < 18 or > 28 kg/m^2^), blood pressure (BP) exceeding 140/90 mmHg or a HR of <50 bpm (beats per minute) or >90 bpm, caffeine consumption of >8 caffeinated drinks/day, or pregnancy/breastfeeding. Data collection occurred during the COVID pandemic restrictions thus Welsh Government informed local regulations regarding COVID‐19 virus transmission avoidance were followed; participants were excluded if they reported a positive lateral flow test on the day of testing or had a positive test or were isolating within the previous 7 days.

### 2.2 Procedure, apparatus and materials

On the day of testing, following the reading of the participant information details, informed consent was obtained. Additional screening was then conducted to ensure that BMI (>18 and <28) and resting BP (not exceeding 140/90 mmHg) and HR (<50 bpm or >90 bpm) were within these healthy ranges. Participants then completed trait mood and anxiety questionnaires. These were the State–Trait Anxiety Inventory (STAI‐Y; Spielberger et al., 1983); Penn State Worry Questionnaire (PSWQ; Meyer et al., 1990); Anxiety Sensitivity Index (ASI; Reiss et al., 1986); hospital anxiety and depression scales (HADS; Zigmond & Snaith, 1983); and modified trait version of the General Anxiety Disorder inventory 7‐items (GAD‐7; Spitzer et al., 2006). The GAD‐7 used a continuous measure of 0–17 (recorded in 0.5 cm divisions), based on ‘Not at all’ = 0 to ‘Nearly every day’ = 17, instead of the original ordinal scale. This allows greater variability to be recorded on this continuous scale.

After completion of trait questionnaires, participants undertook the first of two inhalation conditions which each lasted 20 min in total; the order of delivery of these conditions was air breathing condition followed by the CO_2_ breathing condition (participants were blinded to the order); this took place on the same day, with a sitting rest period of 15–20 min given between inhalation conditions after all data collection had taken place. During the air breathing condition, typical air composition (i.e., 21% oxygen, 78% Nitrogen [N_2_], 0.04% CO_2_) was extracted from room air into a Douglas bag (150 L) and administered via an oro‐nasal breathing mask (Cranlea Human Performance Ltd). Similarly, the CO_2_ inhalation condition was administered via gas cylinder into a Douglas bag and oro‐nasal mask, with a composition breathed of 7.5% CO_2_, 21% O_2_, and 71.5% N_2_. Furthermore, due to the air condition not using a pressurized cylinder and to reduce any expectation bias, white noise was played throughout the duration of both conditions to ensure any experienced auditory cue to each condition was masked.

During each breathing condition, BR was collected using the Biopac M150 Data Acquisition System (MP150‐amplifier and AcqKnowledge‐5.0.2.1 software, Biopac‐Systems, Goleta, CA). BR (breaths per minute – BPM) was collected using the respiration transducer via the wireless respiration (RSP) transmitter at a 2000 Hz sampling frequency and 16‐bit resolution. Automatic rate calculations for BR were used, with the pre‐set ‘BR (for humans)’ being used, with a positive signal peak detect, baseline window width set to 25 ms, a noised rejection of 5% and min (6 BPM) and max (20 BPM) set. The RSP was acquired for the duration of the inhalation period to closely monitor a participant health state. RSP data were only used for analysis during the balance periods of each inhalation (detail of balance periods below). To measure changes in HR and BP an Omron M2 Intellisense Automatic Blood Pressure Monitor was used, with measurements taken just before and immediately after the inhalation sessions. A researcher also stood beside the participant throughout the trial and a crash mat was placed nearby in case the participant felt faint.

During the air and CO_2_ breathing conditions, postural control data were taken as the movement of the centre of pressure (COP) using a force plate (Kistler, 9286BA; Winterthur, Switzerland; 200 Hz, 40–60 mm width and length dimension, 36 mm height from the ground) whilst participants stood in a relaxed standing stance making no sound, with eyes open and arms by their sides; these data were collected at time point 5, 10, and 15 min of the 20‐min inhalation period (5 times in each time point, each lasting 30 s). During all trials, participant stood with socked feet and were asked to fix the gaze at a cross marked 2‐meters away and which was at appropriately eye height. In between each time point, participants were required to sit down.

Subjective measures of emotional state were collected before and after each inhalation period (after manual HR and BP was collected). The measures used were the Positive and Negative Affect Scale (PANAS; Watson et al., 1988) and a modified state version of the GAD‐7 (Spitzer et al., 2006). Completion of the questionnaires took approximately 5 min, after which participants completed another 5 standing trials (post‐inhalation), under the same conditions as during the inhalation period, albeit without the mask inhalation thus breathing normal air.

### 2.3 Data analysis

To ensure that the data were of good quality the raw physiological data were processed and used. The steps taken to pre‐process the BR data from BIOPAC and to reduce any unwanted signals were as follows: BR data were resampled to 100 Hz to reduce data size. A finite impulse response (FIR) bandpass filter was applied with a fixed low filter at 0.5 Hz and high at 1 Hz, with 8000 set for the coefficients (sample rate of 100 Hz) and Q = 0.70700.

All physiology data were checked for outliers and distribution violations, with subject data removed if outliers were >2.5SD from mean, which results in reducing sample for manual HR rate data to *N* = 9. All other data were analyzed using the full sample of 10. Analysis of data from BR and manual HR was conducted using a repeated measures two‐way ANOVA with the factors, *inhalation time* (pre‐ and post‐inhalation‐for manual HR) or during and post inhalation (for BR) and *inhalation type* (Air or CO_2_).

The COP signals calculated by the force plate were not filtered to ensure all relevant information was retained (Riva et al., 2013). Using the COP coordinate position, the following equations were used to calculate sway path length in the medio‐lateral (Equation 1) and anterior–posterior (Equation 2) directions along with the total sway path length (Equation 3):

(1) $$\sum_{n}\left(X_{i+1}-X_{i}\right)$$

(2) $$\sum_{n}\left(Y_{i+1}-Y_{i}\right)$$

(3) $$\sum_{n}\surd {\left(X_{i+1}-X_{i}\right)}^{2}+{\left(Y_{i+1}-Y_{i}\right)}^{2}$$

where *n* = 1499 samples, *X* = medio‐lateral centre of pressure coordinate, *Y* = anterior–posterior centre of pressure coordinate. Non‐linear measures of postural control were used to determine the local dynamic stability of the COP signals. The largest LyE was determined separately for the anterior–posterior and medio‐lateral COP signals using the Rosenstein algorithm (Rosenstein et al., 1993). A state space was reconstructed for each COP signal using the method of time delays (Equation 4):

(4) $$X\left(t\right)=[x\left(t\right),x\left(t+T\right),x\left(t+2T\right),\ldots ,x(t+\left(d_{E}-1\right)T]$$

where *X*(*t*) is the state space vector, *x*(*t*) is the COP time series, *T* is the time delay, and *d*_*E*_ is the embedding dimension. Time delays were calculated separately for each direction from the first of the minimum of the average mutual information function for each trial, average delays for the anterior–posterior and medio‐lateral directions were 67 ± 17 and 79 ± 21 samples, respectively. The d_E_ was determined by global false nearest neighbors analysis and a value of 6 was determined to be appropriate for each direction. For each point in the reconstructed state space all nearest neighbors were identified with a temporal separation equivalent to the mean period of the signal. The Euclidean distance between pairs of nearest neighbors, *d*_*j*_(*i*), was then calculated for each point on the two trajectories. For each pair of points the *d*_*j*_(*i*) was averaged to produce the average divergence as a function of time. The LyE was then determined as the slope of the ⟨ln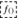 *d*_*j*_(*i*)⟩ versus time plot over the period of 0–0.75 s (Roerdink et al., 2006). An increased LyE represents a greater rate of divergence of neighboring trajectories and is interpreted as a decrease in local dynamic stability.

The statistical analyses for postural control data were performed using the dependent variables of total, medio‐lateral, and anterior–posterior sway path lengths along with the LyE in the medio‐lateral and anterior–posterior directions. Data were calculated separately for each direction as the control requirements differ between directions due to the mechanical constraints of the body. For example, a longer base of support in the medio‐lateral direction compared to the anterior–posterior direction or the action of lower limb muscles primarily in the sagittal plane providing greater movement capacity in the anterior–posterior direction. To compare the change in these measures between the *inhalation type* (Air and CO_2_) and *inhalation time* conditions (time at 5, 10, 15 min and post‐inhalation), separate two‐way repeated measures ANOVA were used.

To explore the impact that a change in breathing rate could have on any change in total sway path length, Pearson's Correlations were performed using the changes between times 5 and 10 min, 10 and 15 min and 15 min and post‐inhalation; this was determined for each breathing conditions.

The questionnaire data collected at baseline, pre and post balance periods were summarized according to standard procedures as described by the authors, except for the GAD‐7 scales. This data measured the distance from the bottom end of a visual analogue line scale in centimeters with higher values indicating more issues with anxiety symptoms. Once all data were summarized, data were checked for outliers and distribution, which indicated no reason to remove any data. For analysis, a similar approach was taken as with the physiology and balance data. A repeated measures two‐way ANOVA was conducted on the GAD‐7 and PANAS (Positive and negative scores), with *inhalation time* (pre and post) and *inhalation type* (Air and CO_2_) as the factors.

All ANOVA analyses used a one tailed alpha set to 0.05, with partial eta^2^ ($\eta_{p}^{2}$) effect sizes calculated and interpreted based on Cohen's guidelines (Cohen, 1988). The Greenhouse–Geisser correction of the degrees of freedom was performed for data violating the assumption of sphericity. Correlations were interpreted using the criteria *r* = .00–.19 “very weak”, .20–.39 “weak”, .40–.59 “moderate”, .60–.79 “strong”, .80–1.0 “very strong” (Evans, 1996). Furthermore, planned Bonferroni‐corrected pairwise comparisons were conducted for all significant main effects and interactions. All analyses and follow‐up test were conducted using IBM SPSS statistics (Version 27) (IBM Corp, 2020).

## 3 RESULTS

### 3.1 Descriptive data and self‐report measures

The means and standard deviations for baseline measures (HR, BP, STAI, ASI, HADS, GAD‐7, PSWQ) can be seen in Table 1. Both the HR and BP are within the previously defined healthy ranges. The self‐report questionnaire baseline data show that the means scores are well below clinical levels.

**TABLE 1:** Means and standard deviations for all physiological and self‐report baseline measures

	*Mean*	*SD*
BMI	21.88	2.41
HR (bpm)	76.30	13.40
BP (mmHg)	120/78.90	9.75/5.71
GAD‐7^a^	2.57	1.60
HADS – anxiety	3.70	2.31
HADS – depression	1.50	1.43
STAI	36.20	7.52
PSWQ	37.00	10.11
ASI	14.30	4.06

Abbreviations: ASI, anxiety severity index; BMI, body mass index; BP (mmHg), blood pressure (millimeters of mercury); GAD‐7, generalized anxiety scale 7 item; HADS, hospital anxiety and depression scale; HR (bpm), heart rate – beats per minute; PSWQ, Penn state worry questionnaire; STAI, state trait anxiety inventory.

^a^ GAD‐7 was measured using a continuous line measure in 0.5 cm divisions, range 0‐17 cm, higher scores demonstrate more anxiety symptoms.

Results of self report measures supported previous CO_2_ challenge studies, replicating, to some extent, increased anxiety and negative mood state as the results of the CO_2_ Challenge (see Table 2 for means and standard deviations). Full ANOVA results are reported in Supporting Information.

**TABLE 2:** Means and standard deviations of manual HR, GAD‐7 and PANAS (NA & PA) by inhalation type (Air and CO_2_) and inhalation time (pre and post)

	Air		CO_2_	
	Pre‐inhalation *M* (*SD*)	Post‐inhalation *M* (*SD*)	Pre‐inhalation *M* (*SD*)	Post‐inhalation *M* (*SD*)
GAD‐7^a^	1.45 (1.20)	1.89 (3.18)	0.91 (0.67)	3.19 (4.45)
PANAS – NA	1.18 (0.13)	1.17 (0.34)	1.08 (0.11)	1.47 (0.51)
PANAS – PA	2.76 (0.64)	2.47 (0.95)	2.55 (0.75)	2.2 (0.88)
Manual HR (bpm)	70.22 (10.32)	68.56 (14.07)	68.22 (9.01)	86.11 (18.09)
BR (bpm)	12.10 (2.72)	10.96 (2.00)	15.00 (1.23)	10.71 (2.70)

	During‐inhalation *M* (*SD*)	Post‐inhalation *M* (*SD*)	During‐inhalation *M* (*SD*)	Post‐inhalation *M* (*SD*)
Total sway path length (m)	0.55 (0.09)	0.55 (0.08)	0.70 (0.22)	0.64 (0.14)

Abbreviations: GAD‐7, generalized anxiety scale 7 item; HR (bpm), heart rate – beats per minute; PANAS, positive and negative affect scale (PA, positive affect; NA, negative affect).

^a^ GAD‐7 was measured using a continuous line measured in 0.5 cm divisions, range 0–17 cm, higher scores demonstrate more anxiety symptoms.

### 3.2 Postural control measures

**FIGURE 1:**
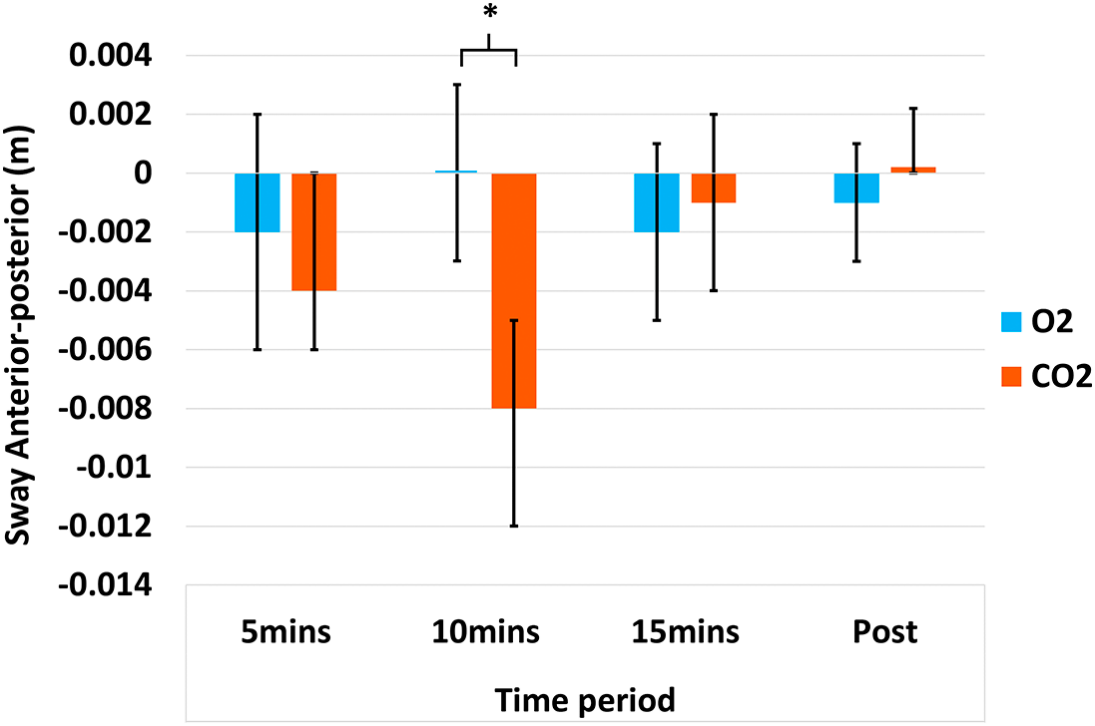
Bar chart (with standard error bars 2±) showing the mean scores in each condition for the 2‐way ANOVA of sway change in the anterior–posterior directions. *p < .05; **p < .01; BPM, breaths per minute; O_2_, typical air; CO_2_, 7.5% CO_2_ enriched air.

Total postural sway path length was significantly higher in CO_2_ inhalation than during the Air inhalation task (*F*[1,9] = 17.19, *p* < .01, $\eta_{p}^{2}$ = 0.67). There was also a significant main effect of *inhalation time* (*F*[1.60,14.44] = 2.82, *p* < .01, $\eta_{p}^{2}$ = 0.24) although the planned pairwise comparisons were not significantly different (*p* > .05). The interaction between *inhalation type* and *inhalation time* was not significant (*F*[1.51, 13.54] = 1.93, *p* = .09, $\eta_{p}^{2}$ = 0.18). There were no main effects for medio‐lateral and anterior–posterior sway path lengths (*p* > .05). However, there was a significant interaction between *inhalation type* and *inhalation time* in the anterior–posterior direction (F[3, 27] = 2.43, *p* = .04, $\eta_{p}^{2}$ = 0.21; see Figure 1), where at 10 min there was greater sway under the CO_2_ condition (*p* = .02).

**FIGURE 2:**
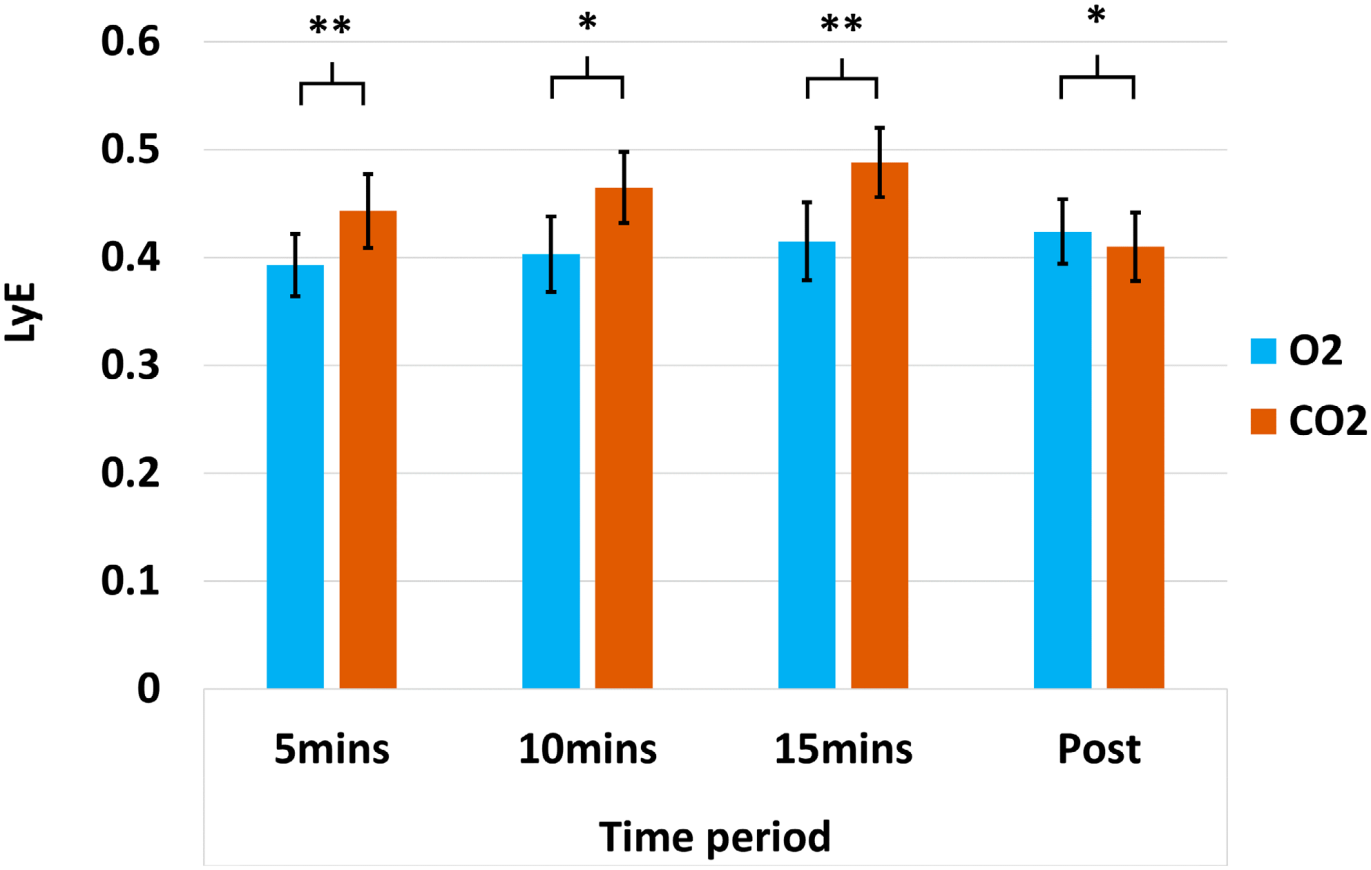
Bar chart (with standard error bars 2±) showing the mean scores in each condition for the 2‐way ANOVA of sway change using the Lyapunov exponent in the y‐axis (LyE). *p < .05; **p < .01; BPM, breaths per minute; O_2_, typical air; CO_2_, 7.5% CO_2_ enriched air.

The LyE was significantly larger during CO_2_ breathing in the anterior–posterior (*F*[1,9] = 18.90, *p* < .01, $\eta_{p}^{2}$ = 0.68) and medio‐lateral directions (*F*[1,9] = 7.04, *p* = .013, $\eta_{p}^{2}$ = 0.44). There was also a significant main effect of *inhalation time* in the anterior–posterior (*F*[1,9] = 2.67, *p* = .034, $\eta_{p}^{2}$ = 0.23) and medio‐lateral directions (*F*[1,9] = 2.86, *p* = .03, $\eta_{p}^{2}$ = 0.24), although planned follow‐up data analysis was not significant *(p* > .05). Furthermore, there was an interaction between *inhalation type* and *inhalation time* in the anterior–posterior direction (*F*[3,27] = 6.87, *p* < .01, $\eta_{p}^{2}$ = 0.43); significantly greater LyE was found during CO_2_ inhalation compared to Air inhalation at 5 min (*p* < .01), 10 min (*p* < .05) and 15 min (*p* < .01), but was decreased post‐inhalation (*p* < .05), see figure 2 (see Table S1 for mean and SD values).

### 3.3 Correlations for change in breathing and total sway path length

For the air breathing, the correlation between the change in breathing rate and total sway path length at 5 and 10 min was moderate and negative (*r*[8] = −.57, *p* = .04). Between 10 and 15 min the relationship it was very weak and non‐significant (*r*[8] = .02, *p* = .48) and between 15 min and post inhalation it was weak and non‐significant (*r*[8] = .26, *p* = .23). During the CO_2_ inhalation period, correlations at 5 and 10 min (*r*[8] = −.09, *p* = .41) and 10 and 15 min (*r*[8] = .08 *p* = .41) were very weak and non‐significant and between 15 min and post inhalation was negative and strong (*r*[8] = −.62, *p* = .03).

### 3.4 Psychophysiological measures and inhalation type

Data collected for the objective measures of heart rate (manual HR) and breathing rate (BR) were analyzed using either pre‐ and post‐inhalation time points (for manual HR) or during‐ and post‐inhalation time points (for BR), see Table 2 for means and standard deviations.

For manual HR there was a significant interaction between *inhalation type* and *inhalation time*, *F*(1,8) = 33.98, *MSE* = 860.44, *p* < .001, $\eta_{p}^{2}$ = 0.81. Pairwise comparisons revealed that in the post‐inhalation period the CO_2_ condition resulted in higher HR (bpm) compared to the Air condition, *p* < .001, while there was no difference in the pre‐inhalation period, *p* > .05. Furthermore, when only looking at the CO_2_ inhalation condition, we find a significant difference in HR between pre‐ and post‐inhalation periods, *p* < .01. There was a significant main effect of *inhalation type* for manual HR, *F*(1,9) = 23.10, *MSE* = 544.44, *p* < .001, $\eta_{p}^{2}$ = 0.74, as well as a significant main effect of *inhalation time* for manual HR, *F*(1,9) = 5.65, *MSE* = 592.11, *p* < .05, $\eta_{p}^{2}$ = 0.41.

**FIGURE 3:**
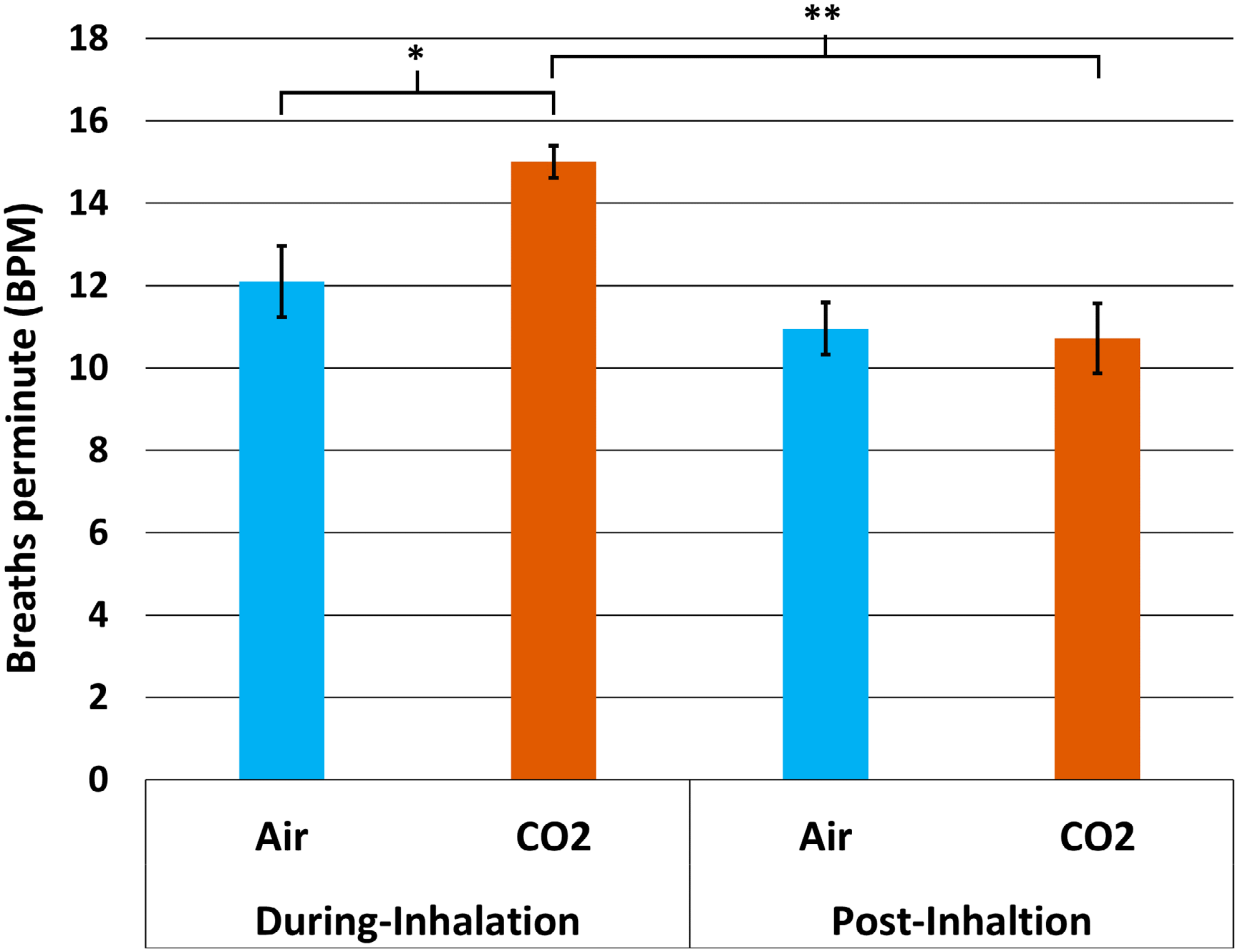
Bar chart (with standard error bars 2±) showing the mean scores in each condition for the 2‐way ANOVA of breathing rate (BPM) *p < .05; **p < .01; BPM, breaths per minute; O_2_, typical air; CO_2_, 7.5% CO_2_ enriched air.

For BR there was a significant interaction between *inhalation type* and *inhalation time*, *F*(1,9) = 6.069, *MSE* = 24.77, *p* < .05, $\eta_{p}^{2}$ = 0.40 (see Figure 3). Pairwise comparisons revealed that in the during‐inhalation period the CO_2_ condition resulted in higher BR compared to the air condition, *p* < .01, while there was no difference in the post‐inhalation period, *p* > .05. There was a significant main effect of *inhalation type* for BR, *F*(1,9) = 8.23, *MSE* = 17.69, *p* < .01, $\eta_{p}^{2}$ = 0.48, as well as a significant main effect of *inhalation time* for BR, *F*(1,9) = 23.82, *MSE* = 73.71, *p* < .001, $\eta_{p}^{2}$ = 0.73.

## 4 DISCUSSION

The aim of this study was to investigate the use of the CO_2_ challenge model of anxiety to better understand anxiety and balance comorbidity. As predicted, the inhalation of CO_2_ (7.5%) enriched air coincided with changes typical of individuals with anxiety (e.g., increased: BR; HR; and negative affect) and reported previously when the model has been used (Ainsworth et al., 2015; Bailey et al., 2007; Garner et al., 2011) However, it is the first to also demonstrate that breathing increased CO_2_ concentrated air can also impact postural control.

Increased total sway path length was shown as a main effect of the CO_2_ condition. Such observation is representative of less control of the postural movement and a greater falls risk (Johansson et al., 2017) and replicates the postural control of individuals with high trait anxiety (Stins et al., 2009). This indicates that the model can experimentally induce trait‐like anxiety in low‐trait anxiety individuals and consequently, postural control adaptations occur to replicate a clinical population.

The current findings suggest this model might through further experiments provide insight into the mechanisms behind anxiety‐balance comorbidity. This is supported by evidence that subjective anxiety during the CO_2_ challenge is positively associated with connectivity between the ventromedial prefrontal cortex and the amygdala as well as having negative associations between the midcingulate cortex and the amygdala (Huneke et al., 2020). The behavior‐related amygdala activity potentially implicates balance‐related networks such as the parabrachial nucleus network (Stins et al., 2009). This is a major brain stem relay centre for visceral information and contains cells that respond to body rotation and position relative to gravity; however, due to the shared connections, it is also involved in the process of anxiety development (Balaban, 2002; Stins et al., 2009). Because of the impact of the anxiety on the vestibular system, there is a greater reliance on sensory input from other systems to maintain upright stance (Balaban, 2002; Brandt & Dieterich, 2020). What is also interesting, is the effect of the CO_2_ inhalation remained beyond the inhalation period. This was shown by the total sway path length collected post inhalation remaining elevated compared to the air condition and is also not different to the data collected during the CO_2_ inhalation period (see Table S1 for mean and SD values). This suggests that the changes in the brain are sustained for a short period of time once normal breathing resumed.

The non‐linear postural control data are also informative regarding the mechanism used to maintain upright stance during the experimental conditions. Sway dynamic stability decreased (i.e., increased LyE) during CO_2_ inhalation and remained consistent in magnitude across the time periods of collection during inhalation. Whilst minimal clinically important change data are not available for these variables in this population, the magnitude of changes found in this study are similar to the magnitude of differences reported between populations with higher and lower balance ability and falls risk (Huisinga et al., 2012; Walsh, 2021) and between higher and lower stability conditions (Ghofrani et al., 2017). These findings suggest the decrease in stability in the current study may correspond to meaningful differences in this population; however, further research is required to determine the minimal clinically important differences in relevant populations. This decreased stability may be the result of more complex, automatic postural control requiring less cognitive resources (Cavanaugh et al., 2007; Donker et al., 2007; Walsh, 2021). The concurrent observation of both increased sway and decreased stability during the CO_2_ breathing trials may therefore be suggestive of lower cognitive resources dedicated to postural control, leading to a reduction in local stability, which would increase falls risk (Walsh, 2021). However, these findings are in contrast to the findings of children with high‐trait anxiety (Stins et al., 2009) who demonstrated lower sway complexity, that is, less automatic postural control. These contrasting findings may be explained by the difference in sample populations. It has been demonstrated that postural control dynamics differ between children and adults (Bisi & Stagni, 2016; Shafizadeh et al., 2021), with children using more active, less automatic postural control mechanisms than young adults (Shafizadeh et al., 2021). The current population of young adults likely had sufficiently robust postural control systems to allow for a decrease in stability, as a result of less active control of posture, without risking a loss of balance.

Literature describes that when an individual has high anxiety, greater attention is given toward a threat which interferes with several other cognitive processes, including how individuals view and respond to their environment (Grant & White, 2016). The act of breathing through a mask may be identified as the source of the anxiety and become the threat to which a greater proportion of their attention is reallocated away from the task of standing. Consequently, a more automated postural control response is experienced, a response replicable of observations made in dual‐task distraction‐based studies (Cavanaugh et al., 2007; Donker et al., 2007; Walsh, 2021). When the mask was removed and normal air breathing resumed, post‐inhalation dynamic stability in the anterio‐posterior sway path was significantly improved following the CO_2_ inhalation condition. This was despite a maintained high overall sway relative to the O_2_ condition and indicates that greater attention was being given to the act of standing. This, however, was not observed in the medio‐lateral direction where LyE remained like the data collected at 5, 10, and 15 min and higher than Air breathing. This may demonstrate that control in the medio‐lateral direction remains relatively autonomous when in an anxious state.

Another explanation for the observed increased in total sway path length during the CO_2_ breathing, is that there was an increased breathing rate in an attempt to remove the CO_2_ from the body, eliciting greater trunk movement. However, the correlation data either showed that the relationship between breathing and sway change to be insignificant or to have a negative association; this negative association would suggest that greater breathing rate change is associated with smaller changes in balance. The non‐significant findings may be due to insufficient statistical power to detect significance. However, assuming the very week correlation size are representative of the wider population, it is likely that this would have had little effect on the differences in sway shown in this study. Furthermore, where significant relationships were shown to be negative this may suggest a mechanism by which potential risk of fall is managed. This may be particularly true for those with the presence of co‐factors such as poorer neuromuscular control or greater anxiety introduced due to the CO_2_ challenge. However, given total sway increased as was expected, it seems that the induced anxiety‐like changes were responsible for the increased sway change observed under the CO_2_ condition. The current findings provide insight into the mechanisms behind anxiety‐balance comorbidity as well as highlighting that experimentally induced anxiety can impact postural control in a similar manner to that seen in clinical populations; this provides further validation of the CO_2_ model of anxiety. However, greater postural control information from different clinical populations is needed (i.e., adults with high GAD) to establish whether changes in non‐clinical participants following the model are accurate. Thus, the lack of data from comparatively aged participants with GAD and other high trait anxiety disorders is a limitation of this study. Furthermore, the use of the model with other populations with low anxiety such as older adults, would be important particularly given that this population already have reduced sway complexity and thus a reduced ability to adapt to the environment (Kedziorek & Blazkiewicz, 2020; Walsh et al., 2021). Finally, it is important to note that the study is a feasibility study with a small sample size and lacking in power, increasing the likelihood of type 1 errors in the context of one‐tailed directional hypotheses. Future research should also explore the impact of different interventions on the postural responses, as well as the study of the cognitive connections that taking place to elicit the symptoms observed in this population.

In conclusion, the study demonstrates the feasibility of using the CO_2_ model to observe change in postural control sway and complexity linked to the occurrence of GAD‐type symptoms, that is, breathing, heart rate and changes in affect. This both supports the validity of the CO_2_ challenge model and the suggested shared neural structure mechanism for comorbidity.

## AUTHOR CONTRIBUTIONS

**Alexander N. W. Taylor:** Conceptualization; data curation; formal analysis; methodology; project administration; resources; supervision; validation; writing – original draft; writing – review and editing. **Daniel C. Low:** Conceptualization; data curation; formal analysis; methodology; validation; writing – original draft; writing – review and editing. **Gregory S. Walsh:** Formal analysis; validation; writing – original draft; writing – review and editing. **Nigel Holt:** Resources; writing – review and editing.

## CONFLICT OF INTEREST

The authors declare that there is no conflict of interest that could be perceived as prejudicing the impartiality of the research reported.

## Supporting information


**Appendix S1:** Supporting information
**Table S1** Means and Standard deviations of balance data at different time point and different inhalation condition

## Data Availability

The data that support the findings of this study are openly available in osf.io at http://doi.org/10.17605/OSF.IO/WZS65, reference number WZS65.
