# Supplementary material for: The impact of anxiety on postural control: CO2 challenge model
Source: Psychophysiology. 2022 Oct 6;60(3):e14192. doi: 10.1111/psyp.14192 (PMC10078562; doi:10.1111/psyp.14192)
Supplement: Supplementary file 1 — Appendix S1: Supporting information Table S1 Means and Standard deviations of balance data at different time point and different inhalation condition [file PSYP-60-0-s001.docx]

**Supplementary materials**

**Additional Methods**

During the inhalation conditions live HR was acquired for the duration of the inhalation period to closely monitor a participant health state. Via a wireless Photoplethysmogram (PPG) transmitter with Photoplethysmogram transducer placed on the left index finger, at 2000 Hz sampling frequency and 16-bit resolution. Automatic rate calculations for HR were used, with the pre-set ‘HR (for humans)’ being used, with a positive signal peak detect, baseline window width set to 100 ms, a noised rejection of 5% and min (50bpm) and max (250bpm) set.

**Data Analysis of additional measures**

The steps taken to pre-process the HR data from BIOPAC and to reduce any unwanted signals were as follows: HR data was resampled to 100 Hz, and an infinite impulse response low and High band pass filter was applied, with low frequency cut-off fixed at 0.5 Hz and high at 10 Hz, Q = 0.70700. Then for each balance period, data was averaged based on the HR rate calculated from automated BIOPAC function, from the 30 second prior to the end marker of a balance period.

Due to unreliable data for the BIOPAC HR data (zero bpm recorded, which was likely due to macro-motion artifacts due to the low intensity motion of moving from seated position to standing on the force plate; Fine et al., 2021; Pietilä et al., 2018) this data was not used in analysis for the purposes of this publication.

Fine, J., Branan, K. L., Rodriguez, A. J., Boonya-Ananta, T., Ajmal, Ramella-Roman, J. C., McShane, M. J., & Cote, G. L. (2021). Sources of Inaccuracy in Photoplethysmography for Continuous Cardiovascular Monitoring. *Biosensors (Basel)*, *11*(4). <https://doi.org/10.3390/bios11040126>

Pietilä, J., Mehrang, S., Tolonen, J., Helander, E., Jimison, H., Pavel, M., & Korhonen, I. (2018, 2018//). Evaluation of the accuracy and reliability for photoplethysmography based heart rate and beat-to-beat detection during daily activities. EMBEC & NBC 2017, Singapore.

**Supplemental Data Analysis**

The PANAS results showed that for the negative affect scales there was an interaction between *inhalation time* and *inhalation type*, *F*(1,9) = 25.71, *MSE* = .40, *p* < .001, *Ƞ_p_^2^* = 0.74. Bonferroni-corrected pairwise comparisons revealed that there was a significantly higher negative affect experienced in pre-inhalation period for Air condition compared to the CO_2_ condition, *p* < 0.05 (see table 2 in main document for means). Importantly, there was a significantly higher negative affect reported in the post-inhalation period for CO_2_ condition compared to the Air condition, *p* = 0.001. Additionally, when comparing negative affect between time points for each inhalation condition, the CO_2_ condition showed a significant increase in negative affect, *p* < 0.01. While the Air condition showed no change between pre- and post-inhalation periods, *p* > 0.05 (see table 2. In main document for all means and standard deviations of self-report data). There was a main effect of *inhalation type* for PANAS negative affect, *F*(1,9) = 5.625, *MSE* = 0.10, *p* < .05, *Ƞ_p_^2^* = 0.39. There was no main effect of *inhalation time* period, *F*(1,9) = 3.188, *p* > 0.05.

There was a main effect of *inhalation time on* PANAS results for positive affect, *F*(1,9) = 8.22, *MSE* = 1.02, *p* < 0.01, *Ƞ_p_^2^* = 0.48 suggesting less positive affect after inhalation was completed. There was also a main effect of *inhalation type* for positive affect, *F*(1,9) = 9.274, *MSE* = 0.58, *p* < 0.01, *Ƞ_p_^2^* = 0.51, suggesting less positive affect as part of the CO_2_ inhalation type. There was no interaction between the factors, *p* > 0.05.

For the GAD-7 there was no main effect of *inhalation time* or *inhalation type*, *p* > .05. While there was a significant interaction, *F*(1,9) = 4.72, *MSE* = 8.349, *p* < 0.05, *Ƞ_p_^2^* = 0.34, however, none of the planned Bonferroni-corrected pairwise comparisons show a significant result in the expected direction, suggesting very little difference in anxiety type symptoms during the experimental conditions.

**Table 1.** Means and Standard deviations of balance data at different time point and different inhalation condition.

|  |  | Air Inhalation | | | | CO_2_ Inhalation | | | | |
| --- | --- | --- | --- | --- | --- | --- | --- | --- | --- | --- |
|  |  | Balance measurement point | | | | Balance measurement point | | | | |
| Balance measure |  | 5min | 10min | 15min | post | | 5min | 10min | 15min | post |
| Sway (A-P) | Mean | -0.0015 | 0.0000 | -0.0018 | -0.0012 | | -0.0042 | -0.0084 | -0.0015 | 0.0002 |
|  | SD | 0.014 | 0.009 | 0.010 | 0.006 | | 0.007 | 0.011 | 0.010 | 0.007 |
| Sway (M-L) | Mean | -0.0001 | 0.0055 | 0.0043 | 0.0032 | | 0.0024 | 0.0036 | -0.0024 | 0.0091 |
|  | SD | 0.014 | 0.017 | 0.027 | 0.018 | | 0.010 | 0.015 | 0.009 | 0.020 |
| Sway (Total) | Mean | 0.5526 | 0.5435 | 0.5453 | 0.5273 | | 0.6519 | 0.7051 | 0.7552 | 0.6166 |
|  | SD | 0.090 | 0.091 | 0.102 | 0.087 | | 0.169 | 0.204 | 0.275 | 0.138 |
| LyE - x-axis | Mean | 0.3640 | 0.4180 | 0.3760 | 0.4300 | | 0.4370 | 0.4560 | 0.4730 | 0.3918 |
|  | SD | 0.040 | 0.065 | 0.043 | 0.080 | | 0.079 | 0.074 | 0.087 | 0.043 |
| LyE - y-axis | Mean | 0.3932 | 0.4031 | 0.4151 | 0.4236 | | 0.4432 | 0.4654 | 0.4882 | 0.4097 |
|  | SD | 0.091 | 0.112 | 0.113 | 0.095 | | 0.108 | 0.105 | 0.102 | 0.101 |
